# Paper-based death record-keeping in Bangladeshi cemeteries: a qualitative exploration of practices and expectations

**DOI:** 10.7189/jogh.15.04093

**Published:** 2025-04-11

**Authors:** Hossain Md Alamgir, Rahman Md Hafizur, Akter Ema, Islam SM Hasibul, Akter Tahmina, Islam Md Shahidul, Ara Tasnu, Maher Manna Ridwana, Tippett Barr Beth A, El Arifeen Shams, Rahman Ahmed Ehsanur, Hossain Aniqa Tasnim, Perkins Janet E

**Affiliations:** 1 Maternal and Child Health Division, International Centre for Diarrheal Disease Research, Bangladesh, Bangladesh; 2 Nyanja Health Research Institute, Salima, Malawi; 3 School of Social and Political Science, University of Edinburgh, Edinburgh, UK

## Abstract

**Background:**

Countries like Bangladesh face significant challenges in effectively registering and tracking deaths within their civil registration systems, which are essential for public health. To improve data collection for public health policy, death record-keeping at burial sites should be enhanced, particularly in areas where burial certificates are issued. With this in mind, we examined the traditional paper-based practices for recording deaths, the perceived significance of these practices, and the associated challenges, expectations, and concerns related to death record-keeping in Bangladeshi cemeteries.

**Methods:**

In 2021, we conducted an exploratory qualitative study involving 25 in-depth interviews with individuals who had lost relatives during the COVID-19 pandemic and key informant interviews with service providers at cemeteries. We complemented these interviews with non-participant observations of burial registration practices in seven cemeteries across urban, peri-urban, and rural settings. We used thematic analysis to interpret the data.

**Results:**

Our findings reveal diverse death record-keeping practices influenced by sociocultural and administrative dynamics, emphasising the necessity of accurate documentation for securing legal rights and social benefits, such as inheritance and welfare. Notable tensions exist between bureaucratic demands and the emotional realities of grieving families, particularly in non-standardised cemeteries, where acquiring death certificates poses challenges. Stakeholders view the anticipated digitalisation of death record-keeping as a transformative opportunity to streamline processes and improve access to information. However, this transition also highlights existing generational and educational disparities in technological skills, alongside ethical concerns regarding data security and user confidentiality.

**Conclusions:**

Our findings showcase the complex interaction between cultural practices, bureaucratic frameworks, and emerging digital technologies in managing death records in Bangladesh. They also emphasise the challenges of modernising traditional documentation methods, as well as the importance of maintaining death records for enhancing civil registration and vital statistics, asserting property rights, and monitoring mortality. A digital system could provide innovative and reliable mortality surveillance from cemeteries.

Countries worldwide use national civil registration and vital statistics (CRVS) systems to track births and deaths. Global health policymakers and programme developers consider death registration to be crucial for monitoring mortality during pandemics and disasters. Yet even outside of moments of crisis, it is considered an important tool for generating data to guide policy development and resource allocation [1]. An estimated 60% of deaths are registered globally, but only an average of 8% are documented in low-income countries [2]. This under-registration has been attributed to a lack of political will, inadequate policy initiatives, resource constraints, and insufficient dedicated staff to support CRVS [3]. Furthermore, if the cause of death is not certified by a physician according to International Classification of Diseases standards, the data are often considered invalid for public health purposes [4]. In addition to informing public health initiatives, improper death registration can cause legal and financial issues associated with death, pensions for widowed spouses, insurance, and inheritance rights [5].

In Bangladesh, approximately 17% of deaths are reported to the national CRVS system [1]. In 2016, the country’s Government estimated that 85% of deaths occurred outside of health facilities, most of which were not registered within the CRVS, resulting in their causes being undetermined [4,6,7].

During the recent COVID-19 pandemic, approximately 30 000 COVID-19 deaths were officially reported in Bangladesh, but the World Health Organization (WHO) estimated the total number of deaths to be nearly five times higher [8]. A well-functioning CRVS system could have supported better reporting of COVID-19-related deaths and the public health response to the pandemic in Bangladesh.

Death registration practices and regulations, including those related to disposing of the deceased, vary widely across countries. Many require official death registration for burial authorisation, which streamlines death registration for CRVS purposes [9,10]. Others, including Bangladesh, have no such legal requirement [9]. Record-keeping at burial sites, if linked to formal systems of death registration, can play a crucial role in strengthening CRVS; for this reason, death registration in cemeteries for public health purposes has been proposed for enhancing death registration in low- and middle-income countries like Bangladesh [11,12]. However, death record-keeping cannot be seen simply as a technical intervention, as it is a complex social process that intervenes in one of the most meaningful phases of human life, with far-reaching social, political, and relational implications [13].

The CRVS has increasingly been accepted as a crucial public health intervention for obtaining accurate, timely data to guide health interventions. Reflecting this logic, the WHO has suggested the registration of births and deaths specifically as ‘the cornerstone of public health and social development planning’ [2]. The COVID-19 pandemic drew increased attention to the limitations in death registration in many countries, including Bangladesh [14,15]. In this context, strengthening death registration in cemeteries could be one approach for strengthening countries’ death registration overall [16,17]. This strategy is not new and has been developing for at least a decade; however, its introduction has been slow in many countries, including Bangladesh. This may be partly due to the limited knowledge of burial sites and systems in these contexts and their social complexities.

With the COVID-19 pandemic exacerbating its limitations, the International Center for Diarrheal Disease Research, Bangladesh (icddr,b) introduced a project to strengthen the CRVS in Bangladesh by improving death record-keeping in cemeteries in 2020. This project used the WHO’s rapid mortality surveillance methodology to collect mortality data from burial sites [18] and, as a further innovation, planned to digitalise death record-keeping in cemeteries to streamline death registration. While the initial objective was to produce actionable data to guide the national public health response to COVID-19, the project sought to strengthen the CRVS in the long term to improve future public health responses to epidemics and health emergencies. Entering the social field of death registration through a global health lens, we sought to explore the current practices and values of death registration in cemeteries and the imaginaries digitalisation inspires in these settings.

## Cemetery landscape in Bangladesh

In Bangladesh, several types of burial sites manage the bodies of the deceased. As a Muslim-majority country, most corpses are buried in cemeteries according to Muslim customs. State-run cemeteries are owned by the local government, with people buried based on the permission of a respective assigned *mohorar* (cemetery manager). Meanwhile, state and non-state-run crematoriums are used by Hindus for cremating the bodies of their deceased. In state-run cemeteries, there are two types of graves: general graves and reserved graves (Table S3 in the **Online Supplementary Document**). General grave users pay BDT 500 (*i.e.* USD 4.19) for two years, after which the grave is reused. Reserved grave users reserve the space for 15 or 25 years by paying a fixed amount decided by the authorities. During this period, family members can reuse the grave by providing BDT 50 000 to bury family members only.

State-run cemeteries in urban areas report deaths directly to the city corporation office, with their managers sending a monthly aggregate report to the social welfare officer of the city corporation official, who is assigned for initial screening and collation. Eventually, the information on deaths in state-run cemeteries is processed and stored by the principal health officer in the birth and death department of the city corporation office. In rural and peri-urban areas, state-run cemeteries report to *upazila nirbahi* officers. Deaths in non-state-run cemeteries are not directly integrated into the national CRVS. Family cemeteries are managed by the family members, and only selected family members can be buried there. Community cemeteries are run by a community-selected committee which ensures renovation and provides verbal permission for burials.

In this study, we explored the current death record-keeping practices in Bangladeshi cemeteries to better understand such practices spanning state, community, and family-managed burial sites. We sought to understand record-keeping practices among cemetery workers, as well as burial site users’ experiences of record-keeping and death registration. We also aimed to explore expectations regarding the potential introduction of digitalised death record-keeping systems.

## METHODS

### Study setting

In Bangladesh, 12 city corporations operate as the local government in urban areas. We conducted this study in state-run cemeteries in Dhaka city corporation areas and a peri-urban area in Chattogram, including six cemeteries in Dhaka city corporation: Uttara-12 cemetery, Uttara-4 cemetery, Uttara-14 cemetery, Banani Budhijibi cemetery, Mirpur Buhijibi cemetery, and Rayerbazar cemetery. We collected the data in homes, cemeteries, and workplaces based on study participants’ preferences.

### Study design

In this exploratory qualitative study, we employed a combination of in-person in-depth interviews (IDIs) and key informant interviews (KIIs), alongside non-participant observation methods. We used IDIs to explore individual experiences and perspectives regarding death record-keeping practices and expectations among those working in burial sites and those who use burial sites. We conducted KIIs to obtain insights from cemetery and city corporation staff who play important roles in death record-keeping. Non-participant observation allowed us to observe and document the practices and dynamics within the cemetery's record-keeping processes. We collected the data between November 2020 to October 2021.

### Study participants

We selected respondents for the study using purposive sampling procedures [19]. We determined the number of participants based on their backgrounds to ensure diverse perspectives and enhance the study's rigour. While we initially predetermined the number of participants, we made adjustments during data collection and initial analysis to better align with our objectives and the experiences of those involved during the COVID-19 period. In total, we conducted 25 IDIs and KIIs (**Table 1**). We selected respondents for IDIs from different social groups who had used cemetery services for deceased family members or relatives, including people facing specific vulnerabilities such as slum residents, homeless individuals, migrant workers, peri-urban and different professionals who have obtained burial experience during the recent COVID-19 pandemic. We conducted KIIs with the following cemetery staff: *mohorar* (*i.e.* cemetery managers), *imam*, cemetery caretakers, and gravediggers. We also conducted KIIs with representatives of the funeral service organisations: Al-Markazul Islami, Anjuman Mufidul Islam, and city corporations. Invitees were informed of the study's purpose, procedures, potential risks and benefits, and how their anonymity would be kept. Those who agreed to participate provided written informed consent before participation.

**Table 1:** Types and number of study participants (n = 25)

	Number of interviews
Vulnerable population*	
Slum resident	2
Migrant	1
Homeless	1
Different geographical location*	
Peri-urban	1
People experience in burial*	
Service holders, workers at non-governmental organisation	5
**Service providers at cemeteries†**	
Senior *mohorar*/*mohorar*	3
*Imam* of the cemetery	2
Caretaker of cemetery	2
Grave digger	3
City corporation staff	2
**Funeral service providing organisations and policymakers†**	
Anjuman Mufidul Islam	1
Al-Markazul Islami	2

IDI – in-depth interview, KII – key informant interview, NGO – non-governmental organisation

*In-depth interview.

†Key informant interviews.

### Data collection

We developed interview guides, field-testing and refining them before use. We maintained field notes of death registration practices and social interactions between cemetery staff and cemetery service receivers. The lead author (MAH), who holds an MSS in anthropology and an MPH degree, conducted all interviews (*i.e.* IDIs and KIIs). Only the interviewer and the respondent were present during the interviews. Based on this, we further revised our semi-structured interview guidelines. All interviews were digitally recorded with the respondents' consent. Each interview lasted 50–60 minutes. The researchers took field notes to document issues that arose during formal and informal conversations. We conducted team debriefings throughout data collection to critically examine the collection procedures and make any necessary changes. Non-participant observation provided insights into death registration practices that went beyond participants' verbal accounts. We conducted an ongoing analysis based on the field notes from the onset of data collection. We maintained a matrix of key findings to ensure sufficient data was gathered to draw meaningful conclusions. We continuously examined the information gathered from the interviews to ensure data saturation, and we ended the data collection when no new themes or insights were emerging. We used this iterative approach to help us decide when there was enough data collected to represent the participants’ viewpoints.

### Positionality and reflexivity

Throughout the study, we practised reflexivity from design to analysis, with team members reflecting on their biases and influences. Before conducting interviews, we built rapport with the respondents through physical communication by maintaining social distancing and using COVID-19 safety measures like face masks, gloves, and personal protective equipment, which helped smooth data collection. Moreover, our affiliation with icddr,b influenced participant perceptions, fostering trust, but potentially biasing responses. We maintained transparency about our backgrounds and motivations, built rapport by explaining study objectives and encouraging open discussion, and used multiple data collection methods. These strategies enhanced the study's credibility and validity by addressing positionality and reflexivity.

### Data analysis

We transcribed the audio recordings and converted handwritten field notes and observation notes into Microsoft Word files in Bangla, which we then converted into .rtf files for uploading to Nvivo, version 12 (Lumivero, Denver, Colorado, USA). We read each interview and observation field note line by line and, based on this preliminary reading, developed an initial codebook (Table S1 in the **Online Supplementary Document**).

Using a reflexive thematic analysis approach, two researchers (MAH and ATH) coded the data and recoded it into themes and sub-themes. The coding tree involved categorising data into the main themes, allowing for systematic organisation and analysis of key findings. Each code represented a specific concept or insight derived from the data, which were then categorised to generate themes.

**Figure 1:**
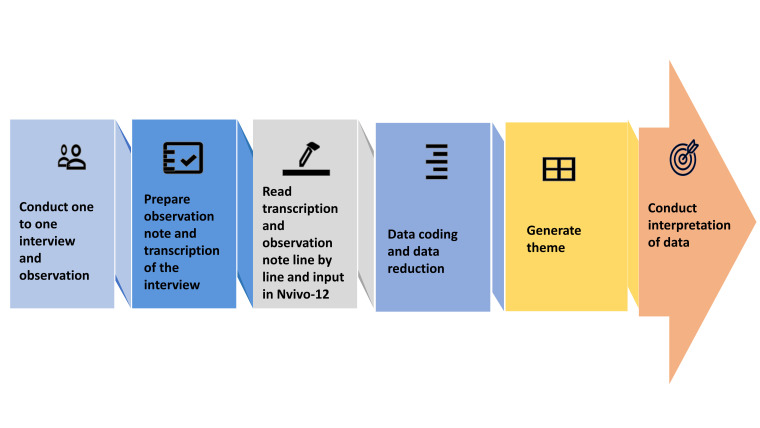
Steps of the data analysis.

In line with our analytical approach (**Figure 1**), we employed inductive strategies to explore the data organically, allowing insights to emerge naturally rather than imposing preconceived notions or expectations on what we anticipate observing within the data. During analysis, we studied uncommon or diverse data and summarised and presented the discoveries according to the context. We used participant quotations (translated to English) to illustrate our findings.

We reported our findings according to the COREQ checklist [20] (Table S2 in the **Online Supplementary Document**).

## RESULTS

### Paper-based record-keeping practices in Bangladeshi cemeteries

We observed a wide variety of death record-keeping practices across cemeteries. State-run cemeteries are managed by a *mohorar* who maintains death records manually. During the burial, families must provide a medical death certificate, supporting documents, and a recommendation letter from a local government representative if the death occurs at home. The *mohorar* records details such as the deceased's information, cause of death, and burial costs, and prepares monthly reports for the social welfare department.

Moreover, public cemeteries rely on verbal orders for record-keeping, while managers maintain records for verification, especially in legal cases. A public cemetery manager explained:

We did not receive any written instructions to maintain death record keeping, we got a verbal order from the birth and death department of the city corporation to keep records and to provide burial-related supporting documents to avoid any criminal case. *(KII, senior* mohorar, *59 years old).*

They also require medical certificates and supporting documents for burials. Monthly reports are submitted to the social welfare department. However, public cemeteries depend on verbal orders for record-keeping, which raises concerns about verification, particularly in legal matters, as a cemetery manager mentioned on the absence of written instructions:

We maintain manual death records as per the verbal instruction of the city corporation authority. However, we have not received any written instruction to keep the medical certificates, the National Identity Card (NID), and other information. Without any [written or verbal] instructions, many of us are not willing to perform proper record-keeping. *(KII, senior* mohorar, *59 years old).*

In contrast, family and community cemeteries lack formal death record-keeping, relying on memory and basic nameplates for identification. Users of the community cemeteries can memorise the grave based on signs that have lasted for a few years. Sometimes family members depend on older family members who remember the deceased person's name and can identify graves. At the gravesite, a small nameplate contains the basic information of the deceased: the name, date of birth, date of death, and address. Besides this, there is no other way to identify the grave and collect death information. Sometimes, a deceased person's family members counted the number of footsteps from a landmark such as a tree or a walled (*pakka*) grave to identify their family member’s grave or counted the number of rows. Moreover, some graves are identified by family members using the nameplate.

There are several hired positions for cemetery management in state-run cemeteries: the *mohorar*, *imam*, grave diggers, and security guards. However, no such positions exist in family-owned or community-managed cemeteries, and there is no designated record keeper. Therefore, family members who bury their relatives in these cemeteries face systematic challenges in securing burial documentation, and then in obtaining death certificates (Table S4 in the **Online Supplementary Document**). A burial service user who used a family cemetery said:

I did not see any death record-keeping system in family cemeteries in rural areas. Although I have seen death registrations in city areas (public cemeteries); however, the formats of the death registration are different across the cemeteries. *(IDI, burial service receiver, 39 years old).*

### Perceived value of death registration

Burial service users expressed the desire to access death records provided by the cemetery, as they enable the issuance of death certificates which are necessary for accessing various benefits. These records, including succession certificates and identifiable documents, help state authorities allocate allowances, relief, and funds to vulnerable individuals, ensuring the proper distribution of resources. Moreover, whenever Bangladeshi expatriates die in Bangladesh, the host country requires a death certificate to verify the death and distribute the benefits to their inheritance. Therefore, family members of migrants are required to obtain death certificates, which are equally valuable for accessing life insurance, banking, and service-related benefits. To access the death certificate, family members must submit the burial receipt collected from the cemeteries during or after the burial. A cemetery manager explained:

A burial receipt is an important document for getting a death certificate. The birth and death department's office requires a burial document. Without documents, there is no way to confirm the death. *(KII, cemetery manager, 50 years old).*

A succession certificate is an important legal document to claim land and other inheritance rights. These certificates can be easily accessible if the family members maintain proper death record-keeping and apply for death certificates. Cemetery users and managers (*mohorar*) explained that local government officials, such as those in city corporations, municipalities (local government offices in urban areas), and union *parishads* (local government offices in rural areas), use death certificates to provide succession certificates to the inheritance landowners.

Succession certifications provide the bureaucratic foundation for determining land inheritance rights. A cemetery manager explained:

Land distribution is a vital issue among the successors. When family members go to claim the land, then they must have required succession certificates for the inheritance land. A death certificate is an important document for distributing the legal portion of land among the siblings. *(KII, cemetery manager, 49 years old).*

Some burial service users highlighted the need for better death record-keeping in cemeteries, especially those who struggled to obtain death certificates. One participant shared his difficulty at the Union *Parishad*, where he needed various documents, including a burial receipt, which he could not provide due to the cemetery's lack of formal records. This forced them to get signatures from the *imam* and a burial attendee, complicating the process. A cemetery service user explained:

I am facing difficulties in getting the death certificate for my father as the community cemeteries do not have the option to maintain the death records. Due to this, I have to collect the signatures from the attendees who have participated in funerals, such as the imams, local leaders, and Union Parishad members. *(IDI, burial service receiver, 39 years old).*

**Figure 2:**
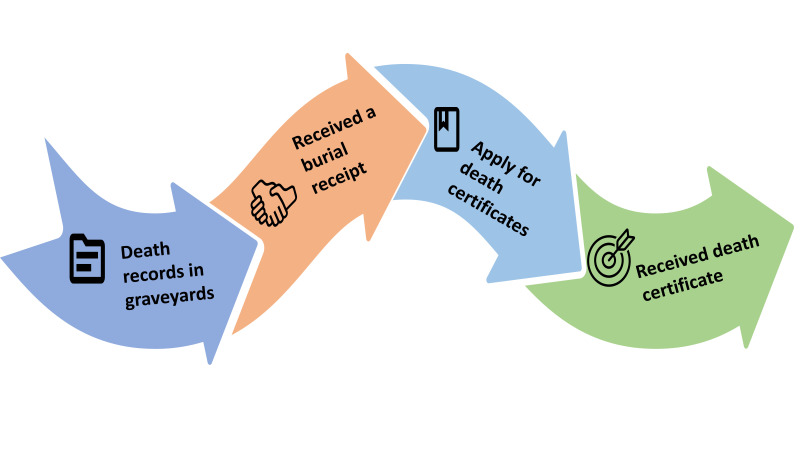
Steps of death records to get death certificates.

Moreover, in rural areas, death registration applications are submitted to the Union Parishad, while in urban areas, they go to the ward counsellor. Family members need to submit three documents: the deceased’s national identity card or birth certificate, the applicant husband’s/wife’s/son’s/daughter’s national identity card or birth certificate, and the burial receipt collected from cemeteries or application with the date and place of burial with the sign of ward member or counsellor for family-owned cemeteries. For those buried in community-based cemeteries, families must obtain the signature of a ward member or counsellor, along with the signature of the cemetery management committee. Those with cemetery receipts do not need to provide extra applications related to show evidence of burial in cemeteries (**Figure 2**).

Based on the burial receipt, family members can apply on an online platform to collect death certificates from the Birth and Death Registration Information System in city areas. Another burial service user who used a family cemetery said:

I live in Dhaka, but my hometown is in Mymensingh. I did not see any death record-keeping system at all in my hometown. I think death record keeping is important to ensure a hassle-free information system. *(IDI, burial service receiver, 37 years old).*

### Death record-keeping encounters in cemeteries

People displayed different reactions when cemetery managers demanded the required documents for burial. Some family members displayed discomfort when providing information required for death registration. Others expressed that they considered the requested information unnecessary (*i.e. oproyojonio*) and became irritable with the extent of the information requested. In some cases, families asked the *mohorar* to identify the nameplate associated with the grave and find the information he sought there (*i.e.* the name of the deceased, date of birth, date of death, and address). They did not see the added value of maintaining death record-keeping beyond this in the cemetery.

Moreover, though death record-keeping is obligatory in city corporation-designated cemeteries, people demonstrated emotional discomfort while completing the death registration during the burial. This was particularly the case when bereaved family members were dealing with a sudden death. A burial service user who used a cemetery for a family member explained:

We are very emotional when we lose family members, close relatives, or friends. During that time, we were not concerned about maintaining the death record-keeping-related formalities. We just overlook it. *(IDI, burial service user, 38 years old).*

There was a COVID-19-dedicated cemetery for COVID-19 deaths. However, family members expressed that the nearest cemetery is preferable due to its easily accessible and desirable burial location. Moreover, cemetery managers and family members stated that family members often hid COVID-19 as the cause of death to obtain permission to bury their deceased family members in a location that meets their desires. In some cases, family members and cemetery managers express that family members managed recommendations for burial approval from the local leaders, such as counsellors and social representatives, by leveraging their social relationships and using social capital, instead of relying on medical certificates as supporting documents on the cause of death in the case of suicide (Table S4 in the **Online Supplementary Document**). A *mohorar* said:

In this place [a renowned cemetery], COVID-19 corpses were not allowed to be buried. Therefore, people tried to hide the cause of death to get permission to bury [their family members] here. *(KII, Senior* mohorar, *59 years old).*

In such cases, bureaucratic demands were negotiated in tension with the social and emotional needs of families grieving a loss.

People who use burial services in family cemeteries without formal death registration face challenges in obtaining death certificates. A burial service user who used a family cemetery said:

We faced problems applying for the death certificate in the city corporation. My relatives were buried in family cemeteries in the city areas, therefore, we did not have any burial registrations. So, we had to collect a written burial certificate from the ward member with the countersignature of the *imam* who led the final prayer (janaja prodan kari). Finally, we collected it and submitted for the death certificate, which is a complete hassle for general people. *(IDI, burial service receiver, 39 years old).*

Those who could not depend on the cemetery for registering the death and acquiring related documentation often had to engage in extensive additional bureaucratic processes and demands. Due to the lack of legitimised death record documentation, family members faced obstacles in collecting the death certificate. Therefore, collecting the death certificate was often a burdensome and lengthy process. Moreover, in some cases, brokers demanded bribes to search multiple registers for specific death information so that families could obtain the death certificate. A migrated vulnerable participant said:

My father died 13 years ago! A few days ago, I went to collect the death certificate but did not find any information about the deceased. For that reason, a broker demanded twelve thousand BDT to find the information. But I have yet to manage this amount of money to collect the death certificate. *(IDI, migrated people, 61 years old).*

### Expectations related to the introduction of digitalisation

Currently, death records are maintained manually, but city corporation representatives, cemetery managers, and service users expect digitalisation to ease workloads and enhance information access. Cemetery users expressed a desire for a platform that helps book vacant graves and anticipated that digital death records could allow instant service access and provide online tools for locating nearby cemeteries and available burial sites. Moreover, cemetery service providers expressed the expectation that digitalisation would enable easier information retrieval, ultimately improving the overall cemetery management process. An *imam* of the cemetery said:

Currently, whenever anyone comes to us to get their required information, it takes time to search the register manually. However, I think online services will make the cemetery service easier for everyone. *(KII,* imam, *50 years old).*

**Figure 3:**
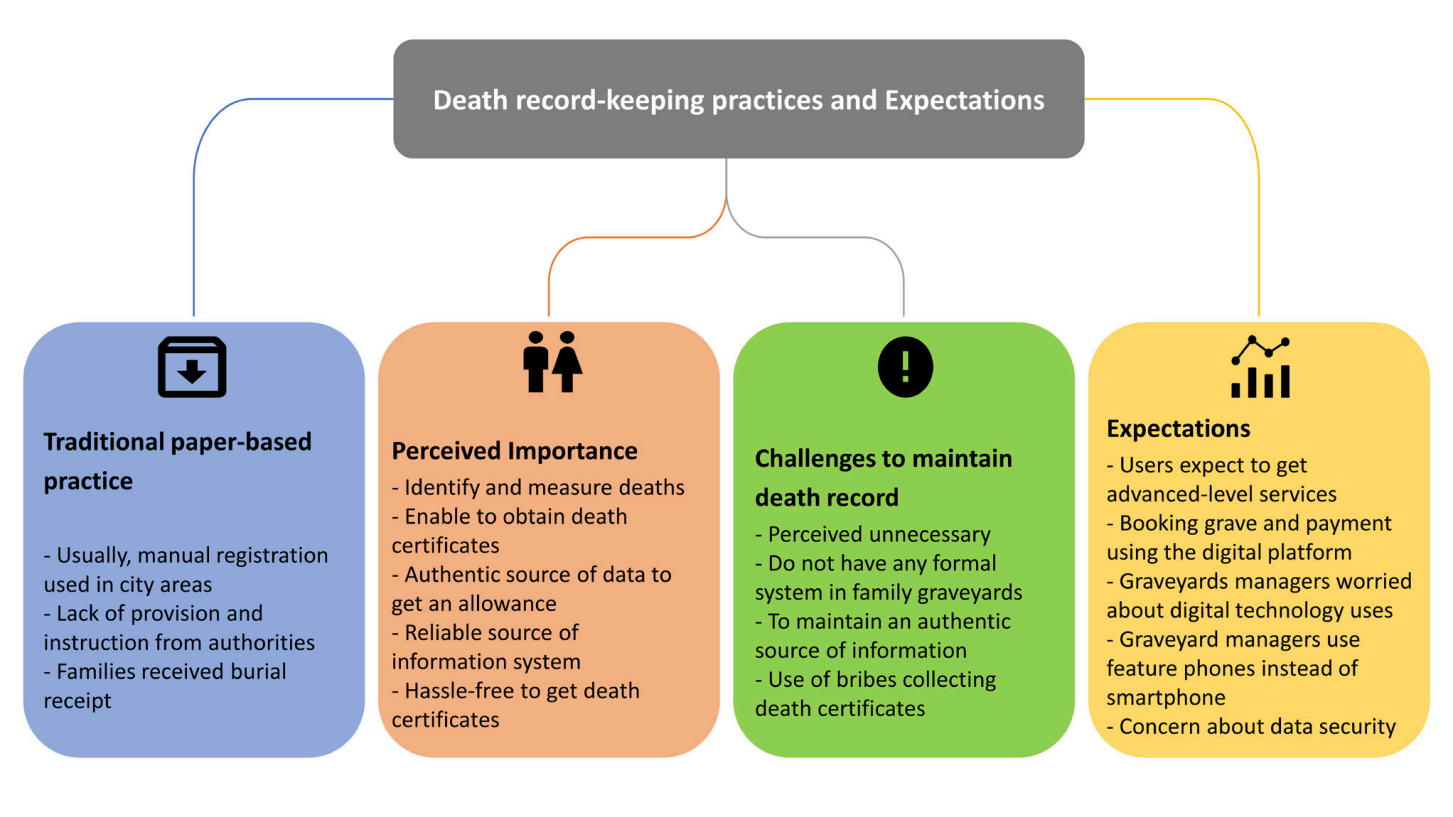
Traditional paper-based record-keeping practice and expectation.

Moreover, users expressed a desire for digital payment options for burial services, as the current cash-based system is seen as outdated. They suggested a digital payment gateway to facilitate easier transactions, especially for expatriates, and a way to streamline the process of selecting grave spaces, booking graves, and purchasing burial-related items, such as bamboo (**Figure 3**). A city corporation staff said:

We could offer a whole package of services, like a ‘supermarket’. The family member of the deceased could get all kinds of services related to burial. We could provide the services using websites and mobile apps. Where users could get access instantly. *(KII, city corporation staff, 44 years old).*

Similarly, city corporation staff expressed optimism that digital death record-keeping would enhance their ability to monitor mortality trends by age, gender, and disease over time. They envisioned the system enabling instant visualisations of causes of death, informing health initiatives, particularly in addressing emerging diseases like dengue and COVID-19. By analysing cemetery-level death reports, they could implement targeted health security measures in high-burden areas. Additionally, some participants suggested that a digital platform could facilitate timely updates to authorities for public health decision-making, allowing for more accurate assessments of national death rates rather than relying on estimates, thereby improving overall public health strategies. A city corporation staff said:

I worked on the urban health project of a city corporation. I feel that we failed to take health initiatives due to the lack of authentic sources of death information. If we have an accurate data source like digital death information, the city corporation could take effective initiatives for diseases like dengue and COVID-19. *(KII, city corporation staff, 44 years old).*

These participants saw digitalisation as having a high potential for leveraging death registration for public health ends. Others suggested that digitalisation could contribute to data accuracy and safety. According to the city corporation staff, using a digitalised server to input and store data could contribute to avoiding user error introduced through manual data input and protect the data from any loss or harm. They imagined everything could be stored on a server and available for further use. A cemetery staff said:

There is a low(er) chance of losing the information if it becomes digital. There is no way to lose, tear, burn, etc. – everything would be stored on the computer. The country's progress in all aspects needs to be digitalised to keep every document authentic and safe. *(KII, cemetery staff, 40 years old).*

However, not all research participants were equally enthusiastic about the possibility of digitalised death records. Some cemetery staff expressed their limitations in using digital devices such as computers and smartphones. Many use feature phones with basic functions for regular communication instead of smartphones and mobile apps. They described themselves as limited in their ability to use technologically advanced devices. During an observation, a *mohorar* showed his feature phone, which he uses to communicate with family members rather than a smartphone. He expressed trepidation using devices as they had limited experience with them. Many *mohorar* explained that their religious-oriented education in a *madrasa* did not prioritise teaching students to use technological devices, and they were, therefore, reluctant to engage in digital death record-keeping. Older *mohorar* nearing retirement expressed even more reluctance to learn computerised technology. A senior *mohorar* said:

We are analogue people! We do our work manually. We do not use a smartphone. Instead, we use a feature phone to receive and forward calls. Most of us are educated in madrasa, and we never try to use the internet as well. *(KII, senior* mohorar, *59 years old).*

Similarly, older, *madrasa*-educated *mohorar* expressed that they were comfortable with manual registration and faced limitations when using computers, smartphones, and digital platforms.

Additionally, while some research participants anticipated that digitalisation could contribute to data safety, others suggested it might compromise security. Some burial service receivers raised concerns about digital data protection. If data were not well protected, it could breach the confidentiality of the users and deceased persons, which would raise an ethical question about using digital platforms. A burial service user who used a cemetery for family members said:

Databases need to be protected from cyberattack. Similarly, the server also needs to run smoothly. If it takes a longer time to reach, then people may lose interest in using it. *(IDI, burial service receiver, 37 years old).*

## DISCUSSION

We found death record-keeping practices varied highly across cemeteries, with the existence of systemised processes in some cemeteries and the absence of registration processes in others. This variability is similar to other settings. In Brazil, for example, Almedia and colleagues collected data from registry offices, cemeteries, and funeral homes and found several unofficial cemeteries that had no logbooks to document burials as people were living in remote areas [21]. In Guinea-Bissau, Fisker and colleagues found that lack of knowledge about its potential benefits was the primary reason for not recording deaths [22]. In our study, much of the differentiation in processes and practices played out across lines of state-run and non-state-run cemeteries. Death registration processes and reporting were robust in state-run cemeteries, while in non-state cemeteries, these processes were inconsistent and ad hoc. However, further exploration of how the state could extend its support to non-state-run cemeteries might provide a more rounded view of possible improvements in the system.

This is perhaps unsurprising, as the state’s interests in registering deaths run beyond solely public health agendas. Indeed, death registration maps onto a longer tradition of what Scott refers to as rendering its population legible [23]. He describes this as a primary objective of a modern state that seeks to know its population and, through this knowledge, control it. Bureaucratic practices, such as those involved in producing CRVS are central to these ambitions. Similar to what Hull describes in his exploration of bureaucracy in Pakistan, documents for registering death in state-run cemeteries become the materiality of bureaucratic objects in daily life [23]. In our study, death record-keeping in state-run cemeteries operated as moments in which bureaucratic objects were enacted, and the power of the state to extend or withhold documentation manifested. In some cases, this power was considered threatening to service users, as it could be used to deny burial or for criminal investigations. Unsurprisingly, many of the participants in our study demonstrated unease with the demands of death record-keeping.

However, our study also revealed the limits of bureaucratic power. Just as state power to bureaucratise death played out in cemeteries, so did the limits of state power. The bureaucratisation of death in our study reveals that, while state power can enforce uniform registration practices in public cemeteries, it does not extend to family-owned and community cemeteries. This indicates a limit to state control, as these private burial sites operate outside the bureaucratic framework imposed by city corporations. That is a reason for financial benefits (*i.e.* bribes) for the death certificate from the deceased family members by the local government staff. As Hull describes in his exploration of bureaucracy in Pakistan, this allows the bureaucracy to become subject to the influence of those beyond the state, such as brokers who demand financial incentives in exchange for navigating the sinuous spaces of bureaucracy and engagement in the production of legitimate documentation [24].

The values inscribed in death registration by public health actors and the state, our study found, were shared by the people navigating the loss of a family member. Our data suggests that for family members of the deceased, death records are principally valuable in terms of how they can contribute to accessing entitlements or settling land disputes; in other words, for the potential of paper value to transform into material value. Land ownership is highly contentious in Bangladesh. Gardner has illustrated that stories of land dispossession create anxiety among people living in Bangladeshi villages [25]. Residents often lack formal ownership documents, further compromising their situation and prospects. Here, *kagojer jomi* is the term used to indicate land acquired legally with formally legitimised documentation. *Kagojer jomi* denotes more substantial ownership rights due to purchase or social legitimisation and was thus a highly desirable resource and stands in contrast to land ownership without paper, which holds ownership in a tenuous position [25]. In our study, family members of the deceased considered death registration valuable when they understood documentation related to registration as imbued with the power to stake claims to land. Registration was also valued for people seeking to make claims to state entitlements, particularly the poor. Helleringer and colleagues demonstrated similar findings in Bangladesh. In their survey, they found people responded that accessing social services and securing an inheritance were the main reasons for reporting recent deaths [1].

In our study, the potential that death record-keeping presented to access resources was the primary draw for people to submit to death registration processes despite their initial reluctance. These findings are similar to those of Marwick and Hargittai in the context of online data sharing in the USA [26]. They found that participants weighed the potential benefits of information sharing, such as convenience and personalisation, against their scepticism, distrust, and fears of discrimination. Their decisions were shaped by the type of information, the context, and the perceptions of state and corporate actors [26]. Our participants also weighed the risks of sharing their personal information with the cemetery – and thereby the state – and the potential benefits of doing so in terms of accessing state entitlements and inheritance.

We found that our participants were looking forward to digitalising death registration. This idea inspired new imaginaries for managing death among those responsible for registering deaths and those who use cemetery services. Digital Bangladesh has been a guiding star in ideas of social and economic development and has inspired the application of cutting-edge technologies to drive economic growth, expand access to education, improve health, reduce poverty, and ultimately enhance opportunities and incomes for Bangladeshi citizens [27,28]. While the drive to create a digital Bangladesh has materialised many societal changes, digitalisation has not been welcomed and experienced uniformly by all. We found that the idea of digitalising death records inspired a range of aspirations: those responsible for death registration imagined that it would ease death registrations processed, making death record-keeping easier and facilitating data sharing with authorities. Cemetery service users thought it might also help them reach their ends. However, some may feel marginalised by the push toward digitalisation, such as the *mohorar* educated in *madrasa* in our study.

Digital technologies achieve beyond what is expected. In a meta-ethnography of point-of-care diagnostics, Perkins and colleagues showcased the interplay between technological innovation and health systems throughout the lifecycle of medical technologies, underscoring the need for socially sensitive evaluation approaches that capture the complex relationships between people, infrastructures, and technologies shaping health system transformation [29]. If digitalisation of death registration is introduced, it will be important to stay attuned to both its anticipated and unanticipated effects. Introducing digital death record-keeping in cemeteries requires motivating older managers to use technology, training existing staff, and providing continuous monitoring and supervision. Key steps include on-the-job training, instruction manuals, and community awareness.

We demonstrated death record keeping in Bangladeshi cemeteries as highly heterogeneous and often ad hoc. These practices are differentially valued by record keepers and those using burial site services. For these actors, the value extends far beyond public health concerns, which directly shape how death record-keeping is enacted. We contend that these considerations must be considered to design socially sensitive interventions for strengthening death registration for public health purposes.

## CONCLUSIONS

Our findings shed light on the webs of health, cultural norms, institutional structures, and power dynamics that extend beyond the logistical aspects of recording deaths. Approaching death record-keeping with a nuanced perspective is crucial to understanding its full role and significance in communities and health systems. The rapidly evolving nature of digital technology could be helpful for policies, access to public service, and proper use of resources by death record-keeping in Bangladesh. However, through digital death record-keeping, we would like to ensure the proper CRVS, digitalised services and benefits, reduced workload, property rights, and updated mortality data.

## Additional Material


Online Supplementary Document
