# Supplementary material for: Paper-based death record-keeping in Bangladeshi cemeteries: a qualitative exploration of practices and expectations
Source: J Glob Health. 2025 Apr 11;15:04093. doi: 10.7189/jogh.15.04093 (PMC11984622; doi:10.7189/jogh.15.04093)
Supplement: Online Supplementary Document [file jogh-15-04093-s001.pdf]

Hossain MA, Rahman HM, Akter E, Islam SMH, Akter T, Islam MS, Ara T, Manna RM, Barr BAT, Arifeen SE, Rahman AE, Hossain AT, Perkins j. Paper-based death record-keeping in Bangladeshi cemeteries: a qualitative exploration of practices and expectations. J Glob Health. 2025;15:04093.

Supplementary Tables:

Table S1: Codes on the death registration on graveyards (cemeteries)

Name	Description
Demographic information	
Age of respondent	
Occupation	
Sex of the participants	
Education	
Income	
Place of burial	
Facilitators of collecting death information using existing death register	
Challenges of collecting death information using existing death register	
Challenges of death certificate collection for community and family graveyard Use	
Challenges of death certificate collection	
Document required during burial	
Enabling factors relating to buried during COVID-19	
Existing burial facilities under city corporation	
The existing register in the graveyard	
Importance of register maintain	
Perceived benefits	
Perceived challenges of register maintain	
Existing staff of graveyard and roles	
Expectations and importance regarding the digital death registration	
Importance of death certificate	
Way of death certificate collection	
Number of known deaths during COVID-19 situation	
Other organizations support burial	
Perceived challenges of online death registration	
Perceived challenges to identifying the grave	
Perception regarding the importance of grave identification	

Name	Description
Perception regarding the importance of online death registration	
Way of preservation of death register or submission of death register	
Way to get permission for burial	
Challenges to getting permission	

Table S2: Consolidated criteria for reporting qualitative studies (COREQ): 32-item checklist

Item No	Guide Questions/Description	Reported on Page #
Domain 1: Research team and reflexivity		
Personal Characteristics		
1. Interviewer/ facilitator	Which author/s conducted the interview or focus group?	Pg 7
2. Credentials	What were the researcher's credentials? E.g., PhD, MD	Pg 7
3. Occupation	What was their occupation at the time of the study?	Pg 7
4. Gender	Was the researcher male or female?	Pg 7
5. Experience and training	What experience or training did the researcher have?	Pg 7
Relationship with participants		
6. Relationship established	Was a relationship established prior to study commencement?	Pg 7
7. Participant knowledge of the interviewer	What did the participants know about the researcher? e.g. personal goals, reasons for doing the research?	Pg 7
8. Interviewer characteristics	What characteristics were reported about the interviewer/facilitator? e.g. Bias, assumptions, reasons and interests in the research topic	Pg 7
Domain 2: study design		
Theoretical framework		
9. Methodological orientation and Theory	What methodological orientation was stated to underpin the study? e.g. grounded theory, discourse analysis, ethnography, phenomenology, content analysis	Pg 7
Participant selection		
10. Sampling	How were participants selected? e.g., purposive, convenience, consecutive, snowball	Pg 7
11. Method of approach	How were participants approached? e.g., face-to-face, telephone, mail, email	Pg 7
12. Sample size	How many participants were in the study?	Pg 7
13. Non-participation Setting	How many people refused to participate or dropped out? Reasons?	Pg 7
14. Setting of data collection	Where was the data collected? e.g., home, clinic, workplace	Pg 6
15. Presence of nonparticipants	Was anyone else present besides the participants and researchers?	Pg 7
16. Description of sample	What are the important characteristics of the sample? e.g. demographic data, date	Pg 6
Data collection		
17. Interview guide	Were questions, prompts, and guides provided by the authors? Was it pilot tested?	Pg 7
18. Repeat interviews	Were repeat interviews carried out? If yes, how many?	N/A

Item No	Guide Questions/Description	Reported on Page #
19. Audio/visual recording	Did the research use audio or visual recording to collect the data?	Pg 8
20. Field notes	Were field notes made during and/or after the interview or focus group?	Pg.8
21. Duration	What was the duration of the interviews or focus group?	Pg 7
22. Data saturation	Was data saturation discussed?	Pg 7
23. Transcripts returned	Were transcripts returned to participants for comment and/or correction?	N/A
Domain 3: analysis and findings		
Data analysis		
24. Number of data coders	How many data coders coded the data?	Pg 8
25. Description of the coding tree	Did the authors provide a description of the coding tree?	Pg 8
26. Derivation of themes	Were themes identified in advance or derived from the data?	Pg 8
27. Software	What software, if applicable, was used to manage the data?	Pg 8
28. Participant checking	Did participants provide feedback on the findings?	N/A
Reporting		
29. Quotations presented	Were participant quotations presented to illustrate the themes/findings? Was each quotation identified? e.g., participant number	Pg 8
30. Data and findings consistent	Was there consistency between the data presented and the findings?	Pg 8
31. Clarity of major themes	Were major themes clearly presented in the findings?	Pg 8
32. Clarity of minor themes	Is there a description of diverse cases or a discussion of minor themes?	Pg 8

Supplementary Figures:

Figure S1: photos of different cemeteries

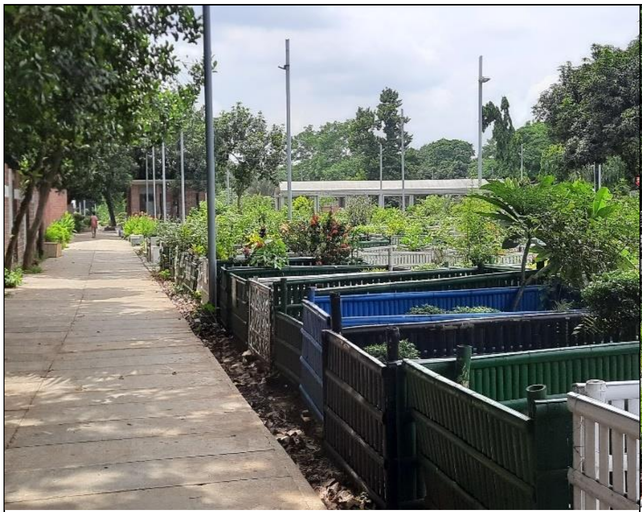

Photos 1: general grave in public cemetery

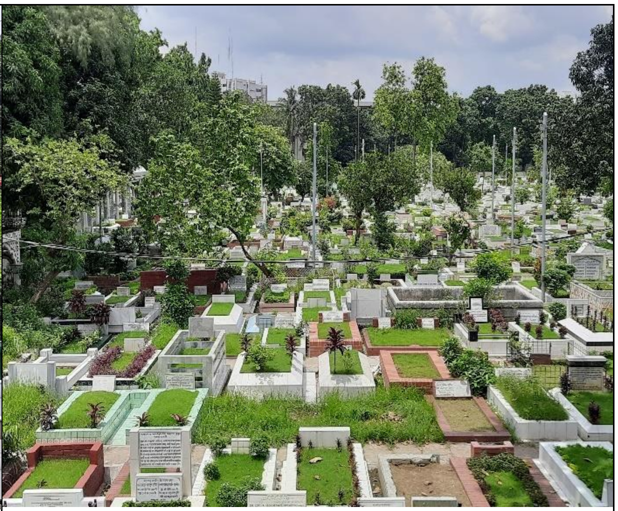

Photos 2: Reserve grave in public cemetery

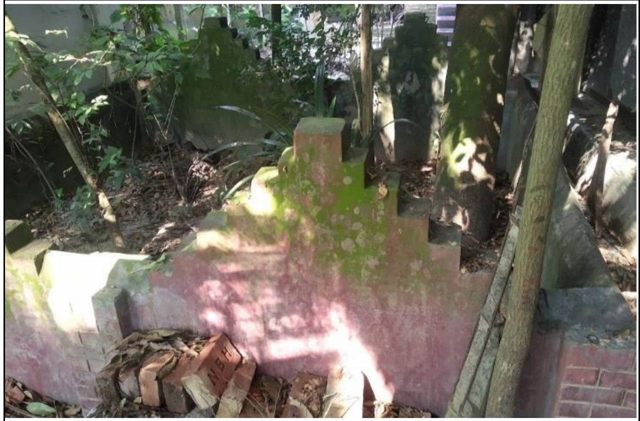

Photos 3: Family-owned cemetery

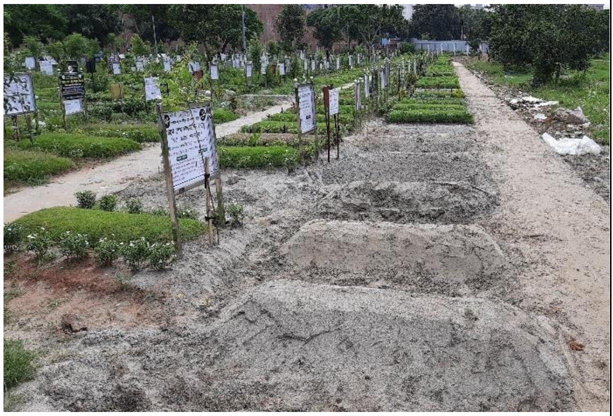

Photos 4: COVID-19 dedicated graves in public cemetery

Figure S2: photos of documents required during burials

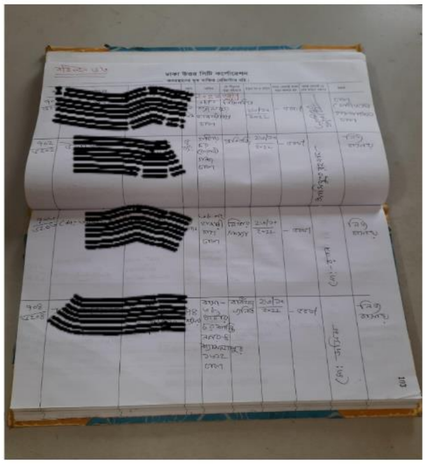 Photos 1: record-keeping in a public cemetery which is regularly monitored	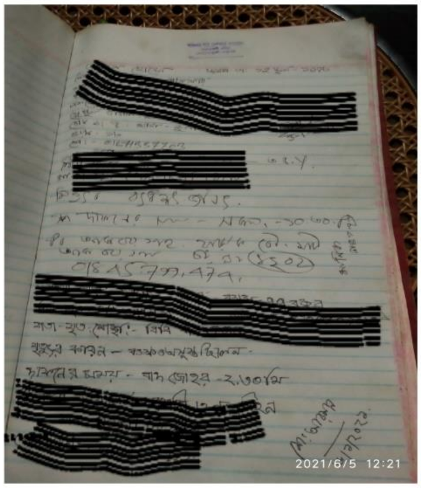 Photos 2: record-keeping in a public cemetery which is not monitored
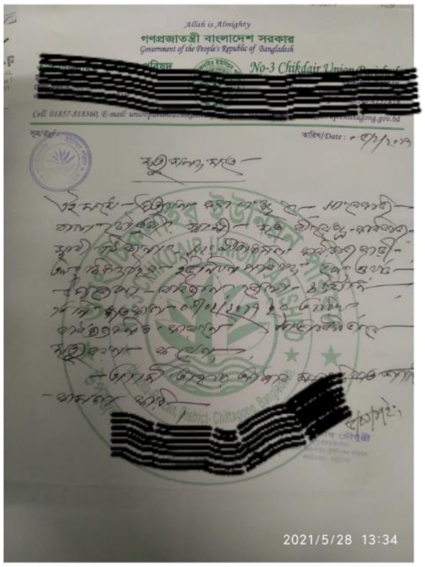 Photos 3: Local leaders' recommendation about deaths	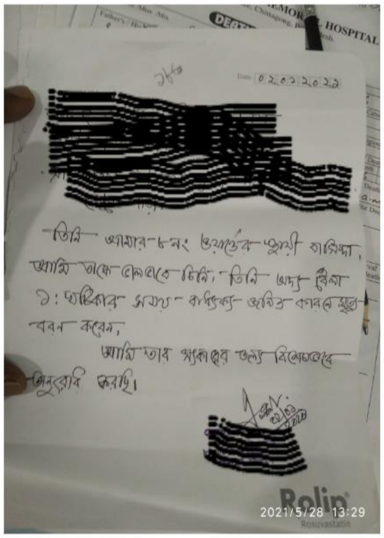 Photos 4: local leader's recommendation about death
